# Impact of Cumulative Unhealthy Sleep Practices in Adolescence on Substance Use in Young Adulthood Estimated Using Marginal Structural Modeling

**DOI:** 10.3389/fnins.2020.00339

**Published:** 2020-04-09

**Authors:** Chia-Yi Ho, Sheng-Hsuan Lin, Meng-Che Tsai, Tsung Yu, Carol Strong

**Affiliations:** ^1^Department of Public Health, National Cheng Kung University Hospital, College of Medicine, National Cheng Kung University, Tainan, Taiwan; ^2^Institute of Statistics, National Chiao Tung University, Hsinchu, Taiwan; ^3^Department of Pediatrics, National Cheng Kung University Hospital, College of Medicine, National Cheng Kung University, Tainan, Taiwan

**Keywords:** insufficient sleep, cigarette use, alcohol use, adolescent, marginal structural model

## Abstract

**Objectives:**

The purpose of this study was to identify the impact of chronic, unhealthy sleep practices in adolescence on substance use in young adulthood. Unhealthy sleep practices in adolescent samples exhibit a bidirectional relationship with substance use. The relationship is further complicated if we consider that confounders such as depression vary over time and are often in response to adolescents’ prior poor sleep practice, which can be addressed by a counterfactual approach using a marginal structural model.

**Methods:**

Data in this study are from the Taiwan Youth Project, a longitudinal study that started in 2000 and surveyed 2,690 7th grade students at age 13. Outcomes include frequency of cigarette smoking and alcohol drinking at age 21. Three unhealthy sleep practices were included in this study: short sleep, social jetlag, and sleep disturbance. We used a marginal structural model with stabilized inverse probability-of-treatment weights to address time-varying confounders in each wave and a total sample of 1,678 adolescents with complete information for this study.

**Results:**

Accumulated waves of sleep disturbance and social jetlag in adolescence were significantly associated with cigarette use in young adulthood. Accumulated social jetlag but not sleep disturbance was also associated with alcohol use in adulthood. Accumulated waves of short sleep were not associated with later alcohol use, but were negatively correlated with cigarette use.

**Conclusion:**

Interventions that aim to reduce the likelihood of substance use in young adulthood should consider confronting unhealthy sleep practices, in particular the discrepancy between bedtimes on school days and weekends and sleep disturbance.

## Introduction

Unhealthy sleep practices, such as sleep insufficiency and social jetlag, are common problems in adolescents related to adverse consequences such as depression, mood disturbances, obesity, and risk-taking behaviors by causing poor judgment and decision-making skills, lack of motivation, and inattention (Owens and Group, 2014; Shochat et al., 2014; Diaz-Morales and Escribano, 2015). Factors that contribute to poor sleep practices in adolescents include but are not limited to increasing media screen time (Twenge et al., 2017), mental health (Lund et al., 2010), and school start time (Gradisar et al., 2011). The definition and measurement of sleep practices relied on using sleep schedule and sleep time to calculate sleep duration (Nascimento-Ferreira et al., 2016). Sleep insufficiency is often defined by short sleep durations, and is sometimes complemented by daytime sleepiness such as daytime napping or weekend oversleeping (Owens and Group, 2014). It is often recommended that adolescents should have 8 h or more for getting sufficient sleep (Owens and Group, 2014). Social jetlag refers to the differences in the timing of sleep that has been interfered with by social schedules, such as the discrepancy between school and free days (Diaz-Morales and Escribano, 2015). The negative impact of poor sleep practices among adolescents is receiving more global attention due to the alarmingly low average sleep time (i.e., less than 8 h) and poor sleep quality (Mesquita and Reimao, 2010; Gradisar et al., 2011; Chen and Gau, 2016; Zhang et al., 2017; Lima and Silva, 2018), especially in Asian samples (Yang et al., 2005; Chung and Cheung, 2008; Liu et al., 2008; Xu et al., 2012).

The initiation of substance use often occurs during adolescence (Montes et al., 2019), which makes adolescence a critical intervention period for substance use. Distinguishing the potential influence of unhealthy sleep practices on substance use can provide insights to reduce substance use among adolescents. Several cross-sectional studies have shown an association between insufficient sleep and substance use in adolescents (O’Brien and Mindell, 2005; Yen et al., 2010; McKnight-Eily et al., 2011). Sleeping less than 8 h per night was associated with smoking and alcohol consumption in a U.S. adolescent sample (McKnight-Eily et al., 2011). Sleep duration at night below the 15th percentile of the study population was associated with high odds of alcohol drinking among Taiwanese adolescents (Yen et al., 2008). Further evidence of the impact of unhealthy sleep practices on substance use was provided by longitudinal studies. For example, a recent longitudinal study identified that erratic sleep/wake and more daytime sleepiness were associated with higher levels of lifetime use of all substances, and that higher evening chronotype tendencies were associated with lower cigarette and higher alcohol use (Nguyen-Louie et al., 2018). Another study found that shorter sleep duration and greater daytime sleepiness were associated with higher odds of later alcohol use (Miller et al., 2017).

Although some longitudinal evidence exists to understand the effect of unhealthy sleep practices on substance use, such a longitudinal relationship, cannot be teased out without considering time-dependent confounders such as depression (Robins et al., 2000). For example, depression in adolescents is also associated with later substance use (Conway et al., 2016) and can be affected by prior poor sleep practice (Owens and Group, 2014). Other confounders such as academic performance and self-rated health were also associated with both sleep practices and substance use (Diego et al., 2003; Curcio et al., 2006; Ortiz-Hernandez et al., 2009; Conklin et al., 2019). A failure to control for confounders, which are affected by prior treatment, and a risk factor for the outcome such as substance use in our study, would introduce a biased estimate of the association (Robins et al., 2000).

**FIGURE 1:**
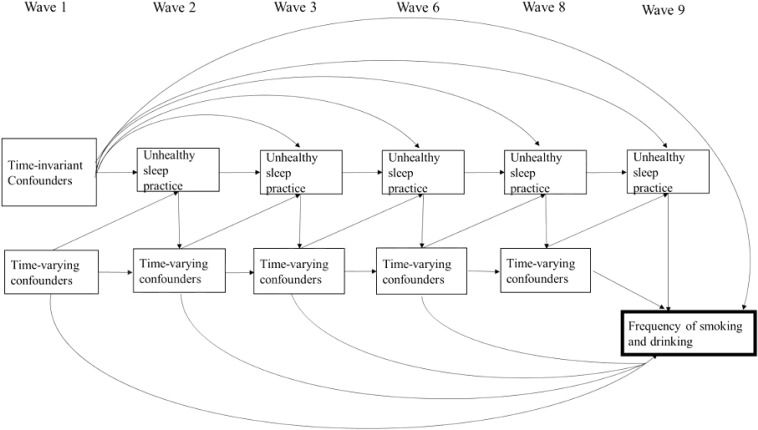
Conceptual framework denoting the relationships between cumulative unhealthy sleep practices in wave 2 to 9 and frequency of smoking and drinking at age 21. Sleep practices of adolescents were affected by sleep practices and time-varying confounders in the previous waves. Time-invariant confounders included gender, level of urbanization of school, township type of school location, pubertal timing, expected academic achievement, and father’s ethnic origin. Time-varying confounders included depression, self-related health, academic achievement, attending cram school, smoking and drinking status, and living with parents or not. Time-varying confounders not only affect unhealthy practice for the next wave, but also the following wave, which were omitted for the clarity of the graph.

In this study, we used a marginal structural model to address the issue of time-varying confounders. Marginal structural models were used for observational data to estimate the causal effects of an exposure that changes with time. The covariates in this model also changed with time and could play a role both as confounders and mediators (Robins et al., 2000). The application of a marginal structural model has been used in several research fields such as epidemiology, criminology, and medicine (Petersen et al., 1983; Yi et al., 2009; Lin and Yi, 2015). We aim to examine the impact of cumulative sleep insufficiency in adolescence on cigarette smoking or alcohol drinking in young adulthood in a sample of Taiwanese adolescents that have been followed for 17 years. A hypothesized conceptual framework is depicted in Figure 1 denoting the relationships between cumulative unhealthy sleep practices and the frequency of smoking and drinking at age 21. We also tested various types of unhealthy sleep practices that resulted in sleep insufficiency, including short duration of sleep, social jetlag, and sleep disturbance. We hypothesized that chronic, unhealthy sleep practices have an impact on cigarette smoking and alcohol drinking in young adulthood after adjusting for time-varying confounders such as depression, academic achievement, and self-rated health. We also compared the estimates from conventional regression analyses with marginal structural models.

## Materials and Methods

### Participants

Data in this study were from the Taiwan Youth Project (TYP) (Yi et al., 2009). TYP was a longitudinal study that started in 2000 and surveyed two cohorts of 7th (J1) and 9th (J3) grade students from northern Taiwan (Yi et al., 2009). TYP used a multistage-stratified and class-clustered method to randomly select 40 schools and 81 classes in each grade (Yi et al., 2009). Only the J1 cohort, 2,690 7-grade participants, was used for this study. For this study, we used data from waves 1 (year 2000, age = 13, *n* = 2,690), 2 (year 2001, age = 14, *n* = 2,683), 3 (year 2002, age = 15, *n* = 2,664), 6 (year 2005, age = 18, *n* = 1,826), 8 (year 2007, age = 20, *n* = 1,739) and 9 (year 2009, age = 21, *n* = 1,875). We excluded students who had ever smoked or drank alcohol in 1st wave (*n* = 206) and those who failed to complete the questions about frequency of cigarette use and alcohol use at wave 9 (*n* = 806). Finally, the sample used for analysis in the present study was 1,678 participants. The excluded sample was not statistically different in gender, level of urbanization of school, township type of school location and father’s ethnic origin compared to the 1,678 participants used for the analysis in this study. The study protocol was approved by the Human Ethics Committee at the National Cheng Kung University Hospital (B-ER-107-028).

### Measures

#### Dependent Variables (Cigarette and Alcohol Use at Wave 9)

Participants were asked how many packs of cigarettes they smoked in the past week on a scale from never to at or above 7 packs and how many times they drank alcohol in the past month on a scale from never to at or above 7 times at age 21. The frequency of substance use was treated as a continuous variable.

#### Independent Variable (Unhealthy Sleep Practices)

Three unhealthy sleep practices were assessed in this study: short sleep, social jetlag, and sleep disturbance; all of these were measured at wave 2, 3, 6, 8, and 9. In each wave, the participants were asked what time they went to bed and got up on weekdays and what time they went to bed and got up on weekends. A weekly average was calculated first by adding up the total hours of sleep within a week and then dividing by seven. We defined short sleep as less than an average of 8, 7, or 6 h per night in a week. We used various cutoffs because the definition of sleep insufficiency varies by culture and time period (Nascimento-Ferreira et al., 2016). Although it is often recommended to have 8 h or more for getting sufficient sleep for adolescents (Owens and Group, 2014), since the average sleep time was quite low in several Asian samples (Yang et al., 2005; Chung and Cheung, 2008; Liu et al., 2008; Xu et al., 2012), we decided that the cutoff should not only consider the recommended 8-h but also included other cutoffs, such as 7- and 6-h, in the analysis. Cumulative short sleep was the sum of waves of short sleep from wave 2 to 9. Social jetlag was calculated by subtracting weekday bedtime from weekend bedtime, a definition used by previous studies (Lin and Yi, 2015; Jankowski, 2017). A positive score indicated that students went to bed later on the weekends. Dichotomized social jetlag was defined with three cutoffs: at or above 2, 1, or 0.5 h. Cumulative social jetlag was a sum of waves of social jetlag from wave 2 to 9. Finally, we used three items to capture the students’ sleep disturbance: insomnia or difficulty falling asleep, waking early and unable to go back to sleep, and disturbed night sleep or waking up often during the night. The response categories ranged from “it does not happen” (0) to “it happens to me and is very serious” (4). The sleep disturbance items were similar to those described in the Sleep Quality Index (SIQ) (Cronbach’s α = 0.60–0.71) (Yi et al., 2009; Lin and Yi, 2015). Sleep disturbance for each wave was indicated by a sum score. Cumulative sleep disturbance was the sum of waves of sleep disturbance from waves 2 to 9.

#### Time-Invariant Covariates (Wave 1)

##### Gender

Students were either male or female.

##### Level of urbanization of school

There are geographical and urbanity differences in Taiwan in prevalence of substance use behavior (Chen et al., 2017). School location was on a scale of 1 (rural/Yilan County) to 3 (urban/Taipei City) based on the stratification of Taiwan administrative districts (Yi et al., 2009).

##### Township type of school location

The township type of school location was classified on a scale of seven types of townships: core city, general city, emerging towns, traditional industrial towns, general towns, aging towns, or remote area (Yi et al., 2009).

##### Pubertal timing

The Pubertal Development Scale (PDS) was used to measure the participants’ pubertal timing (Petersen et al., 1983). The PDS used five items: height spurt, body hair development, skin changes, breast growth/deepening of voice, and menarche/facial hair development. Except for menarche, which was a dichotomous item (“yes” or “no”), all other items were rated using a 4-point Likert scale. The internal consistency of the PDS is acceptable in boys (α = 0.68–0.78) and girls (α = 0.76–0.83) (Petersen et al., 1988), and α = 0.681 for boys and α = 0.713 for girls in Taiwan sample, which included 1,378 boys and 1,312 girls (Tsai et al., 2015b). Based on standardized same-gender PDS scores, the participants were classified into three pubertal timing groups: early puberty (more than 1 standard deviation [SD] above), on-time puberty (within 1 SD either way), and late-puberty (more than 1 SD below) (Negriff et al., 2011; White et al., 2012; Tsai et al., 2015a, b).

##### Expected academic achievement

Expected academic achievement may indicate the level of parental monitoring (Pinquart, 2016), which may affect adolescents’ bedtime (Gunn et al., 2019) and substance use (Smith et al., 2017). Students were asked the educational level they desired if there were no restrictions. We dichotomized the answers into “college graduate or above” and “below college.”

##### Father’s ethnic origin

Father’s ethnicity was categorized as Weinan Islanders, Hakka, mainlanders, original residents, or others.

#### Time-Varying Covariates

##### Depression (waves 1, 2, 3, 6, and 8)

A short version of the Symptom Checklist-90-Revised (SCL-90-R) consisted of seven items: headache, lonely, depressed, weakness in certain parts of the body, numbness/tingling pain in certain parts of the body, feeling like the throat is clogged, and insomnia (Derogatis, 1983; Lin and Yi, 2015). Adolescents were asked whether they had experienced these symptoms in the past week and how serious the experience was in the past week on a scale of 0 (no) to 4 (yes, very serious). An average score of 6 items (not including insomnia) divided the adolescents into four groups: 0, at or less than 1, at or less than 2, and more than 2.

##### Self-related health (waves 1, 2, 3, 6, and 8)

Adolescents were asked how they felt about their health; response categories ranged from 1 (excellent) to 5 (very bad). We classified them as “good” when they chose excellent, very good or good, and “bad” when they chose not very good or very bad.

##### Academic achievement (waves 1, 2, 3, and 6)

Students were asked to self-report their ranking in class. We divided them into two group: rank 1st to 5th and others.

##### Attended cram school (waves 1, 2, 3, and 6) or had a job (waves 6 and 8)

Cram schools are private after-school programs for students to achieve better academic performance. Students were asked whether they were attending cram school or had been working in the past year.

##### Self smoking and drinking status (waves 2, 3, 6, and 8)

Students were asked whether or how often they smoked or drank in the past year. Answers were categorized as yes if they have smoked or drank and no if not.

##### Peer smoking and drinking status (waves 2, 3, 6, and 8)

Students were asked whether or how often their peers smoked or drank in the past year. Answers were categorized as yes if they have smoked or drank and no if not.

##### Living with parents (waves 1, 2, and 3)

Answers were categorized as living with parents or not.

### Statistical Analysis

We first used conventional linear regression analyses, controlling for the confounders (includes time-invariant covariates and time-varying covariates at wave 1, and the other two sleep variables) to analyze the relationship between accumulated waves of unhealthy sleep practices and the frequency of cigarette and alcohol use in wave 9 (Model 1).

We then conducted an analysis using marginal structural logistic regression models to examine the frequency of cigarette smoking and alcohol use in young adulthood (21 years) as a function of cumulative unhealthy sleep practices during adolescence and time-invariant covariates. Adjustment for time-varying covariates was achieved through the use of inverse probability-of-treatment weights (IPW) (Robins et al., 2000). We calculated the probability of the unhealthy sleep practices in subsequent waves by using logistic regression. The inverse of this probability was used to weight each respondents’ contribution to a pseudo-population in which time-varying covariates were balanced in expectation across the healthy sleep practices.

Because the variables were either nominal or ordinal, we used “indicator variable analysis” to deal with item non-response missing data (Faries et al., 2014). We substituted these missing data with a number to represent an additional category of each variable. We considered participants who were lost-to-follow-up as exposed to another treatment. In wave 1 and wave 9, there were 1,678 students; 5, 15, 353, and 295 students were attritions in wave 2, 3, 6, and 8, respectively. Using inverse probability attrition weighted (IPAW) estimation, the probability of lost-to-follow-up was considered the same (Goldstein, 2009).

We used marginal structural models to estimate the effects of each additional wave of unhealthy sleep practices on the frequency of cigarette smoking and alcohol use in wave 9 (Figure 1). That is, we used stabilized IPWs (Model 2) and further added the other two sleep variables as the time-varying covariates to calculate adjusted stabilized IPWs (Model 3). The IPTWs were multiplied by IPAWs to get a correct weight, and the marginal structural models were fitted using a conventional linear regression model that did not include the time-varying covariates as controls. All of the analyses were computed by SAS Enterprise Guide, version 9.4, computer software.

## Results

Tables 1, 2 present descriptive statistics for the time-invariant and time-varying sample characteristics. Half of the participants were male and at wave 1 when they aged 13, 58.6% were at late puberty and 34.6% were categorized as on-time puberty (Table 1). At wave 9, when they aged 21, participants smoked 0.57 packs of cigarettes per week on average and drank 0.92 times per month (Table 2). The majority of participants did not smoke or drink: 85.2% did not smoke and 60.1% did not drink alcohol at wave 9.

**TABLE 1:** Time-invariant sample characteristics.

	At Wave 1 (*n* = 1678)
Variables	%
**Gender**	
Male	50.4
Female	49.6
**Level of urbanization of school**	
Taipei City	37.7
New Taipei City	37.9
Yilan County	24.4
**Township type of school location**	
Core city	48.2
General city	27.5
Emerging towns	13.5
General towns	8.1
Aging towns	2.6
**Pubertal timing**	
Early puberty	6.8
On-time puberty	34.6
Late-puberty	58.6
**Expected academic achievement**	
At or under junior college	24.3
At or above college	74.3
**Father’s ethnic origin**	
Weinan Islanders	77.5
Hakka	8.1
Mainlanders	11.9
Original residents	0.8
Others	1.6

**TABLE 2:** Time-varying sample characteristics.

	Wave 1	Wave 2	Wave 3	Wave 6	Wave 8	Wave 9
	(n = 1,673)	(n = 1,673)	(n = 1,673)	(n = 1,673)	(n = 1,673)	(n = 1,673)
	
		%	%	%	%	Mean ± SE or %
**Independent variables**						
Sleep disturbance (sum score > 0)		44.6	55.8	43.4	59.9	53.3
Short sleep						
<8 h		50.5	64.9	71.8	48.4	45.2
<7 h		14.5	27.5	34.5	18.6	15.3
<6 h		3.1	7.6	8.5	3.4	3.5
Social jetlag						
≥0.5 h		83.7	78.5	65.4	68.4	65.1
≥1 h		64.7	58.5	46.9	55.8	49.7
≥2 h		26.1	21.4	15.3	18.8	16.0
**Dependent variables**						
Cigarette smoking (packs/week)						0.57 ± 1.63
Alcohol use (times/month)						0.92 ± 1.49
**Time-varying covariates**						
Depression, %						
0	33.2	27.6	21.5	24.7	22.0	
At or less than 1	58.1	62.2	61.3	59.1	59.1	
At or less than 2	7.9	9.7	16.1	15.2	17.1	
More than 2	0.7	0.5	1.14	1.1	1.8	
Self-related health (bad, %)	13.6	6.2	6.6	7.0	14.8	
Academic achievement (within the top five grades, %)	17.0	17.4	17.9	17.0		
Attended cram school (Yes, %)	67.4	58.7	54.1	43.8		
SELF smoking and drinking status (Yes, %)		7.8	5.6	29.5	32.3	
PEER smoking and drinking status (Yes, %)		0.84		31.6	42.9	
Living with parents (Yes)	89.2	88.8	87.7			
Had a job (Yes, %)				14.8	46.0	

At wave 1, sleep disturbance was significantly correlated with social jetlag and short duration of sleep; short duration of sleep was not correlated with social jetlag. There are similar proportions of participants suffering sleep disturbance in waves 2, 3, 6, 8, and 9 (43.4–59.9%). In most waves, half of the participants had short sleep defined as less than 8 h. Participants (ranging from 14% to 34.5%) had short sleep defined as less than 7 h. About half of the participants had social jetlag greater than or equal to 1 h (46.9–64.7%).

### Sleep Disturbance

In the conventional linear regression model (Model 1 in Table 3), accumulated waves of sleep disturbance could increase the times of alcohol use per month (β = 0.071, 95% CI = 0.020, 0.122), but they were not associated with the frequency of cigarette smoking. In the marginal structural models, sleep disturbance significantly increased the packs of cigarettes smoked per week (stabilized weights in Model 2: β = 0.076, 95% CI = 0.019, 0.133; adjusted stabilized weights in Model 3: β = 0.074, 95% CI = 0.017, 0.130), but were not associated with times of alcohol use per month (Table 3).

**TABLE 3:** Accumulated waves of sleep disturbance and frequency of smoking and drinking.

	Packs of cigarette smoking per week	Times of alcohol use per month
	
	β (95% CI)	β (95% CI)
Model 1	0.043(−0.010,0.095)	0.071(0.020,0.122)**
Model 2	0.076(0.019,0.133)**	0.045(−0.008,0.097)
Model 3	0.074(0.017,0.130)*	0.045(−0.007,0.097)

Model 1: conventional linear regression analyses, controlling for the confounders including time-invariant covariates and time-varying covariates at wave 1, and the other two sleep variables. Model 2: used stabilized inverse probability attrition weighted (IPTW). Model 3: Model 2 further added the other two sleep variables as the time-varying covariates to calculate adjusted stabilized IPTW. *p < 0.05, **p < 0.01.

### Short Duration of Sleep

In the conventional linear regression model (Model 1 in Table 4), accumulated waves of short sleep—no matter whether the sleep duration was less than 8, 7, or 6 h—were not associated with the frequency of cigarette smoking and alcohol use. However, using IPTW, when the sleep duration was less than 8 h, each additional wave had decreased packs of cigarette smoking per week (stabilized weights in Model 2: β = −0.106, 95% CI: −0.17, −0.042; adjusted stabilized weights in Model 3: β = −0.107, 95% CI: −0.171, −0.043), but had no significant effect on alcohol use. When the sleep duration was less than 7 h, each additional wave had decreased packs of cigarette smoking per week (stabilized weights in Model 2: β = −0.081, 95% CI: −0.159, −0.002), but had no significant effect on alcohol use. Sleep duration less than 6 h for each additional wave increased the times of alcohol use per month after adjusting for the other two unhealthy sleep practices as time-varying confounders (adjusted stabilized weights in Model 3: β = 0.196, 95% CI = 0.035, 0.357), but had no significant effect on cigarette smoking (Table 4).

**TABLE 4:** Accumulated waves of short sleep and frequency of smoking and drinking.

		Packs of cigarette smoking per week	Times of alcohol use per month
		
		β(95% CI)	β (95% CI)
<8 h	Model 1	−0.008(−0.063,0.046)	0.018(−0.035,0.071)
	Model 2	−0.106(−0.170,−0.042)**	−0.009(−0.067,0.049)
	Model 3	−0.107(−0.171,−0.043)**	0.004(−0.055,0.062)
<7 h	Model 1	0.010(−0.059,0.079)	0.036(−0.031,0.103)
	Model 2	−0.081(−0.159,−0.002)*	0.046(−0.028,0.121)
	Model 3	−0.064(−0.146,0.017)	0.053(−0.023,0.129)
<6 h	Model 1	0.031(−0.103,0.165)	0.020(−0.110,0.149)
	Model 2	−0.056(−0.222,0.111)	0.116(−0.037,0.268)
	Model 3	−0.048(−0.227,0.130)	0.196(0.035,0.357)*

Model 1: conventional linear regression analyses, controlling for the confounders including time-invariant covariates and time-varying covariates at wave 1, and the other two sleep variables. Model 2 : used stabilized inverse probability attrition weighted (IPTW). Model 3: Model 2 further added the other two sleep variables as the time-varying covariates to calculate adjusted stabilized IPTW. *p < 0.05, **p < 0.01.

### Social Jetlag

By the marginal structural models, accumulated waves of social jetlag increased the packs of cigarette smoking per week and the times of alcohol use per month, regardless of whether the differences of bedtime between weekday and weekend were longer than 0.5, 1, or 2 h. However, some of the effects could not be observed in the conventional linear regression analysis. We also found that the larger social jetlag was, the higher the frequency of cigarette smoking and alcohol use (Table 5).

**TABLE 5:** Accumulated waves of social jetlag and frequency of smoking and drinking.

		Packs of cigarette smoking per week	Times of alcohol use per month
		**β (95% CI)**	**β (95% CI)**
≥0.5 hours	*Model*1	0.047(−0.015,0.110)	0.018(−0.042,0.079)
	*Model*2	0.141(0.064,0.218)***	0.095(0.027,0.163)**
	*Model*3	0.138(0.061,0.215)***	0.100(0.031,0.169)**
≥1 h	*Model*1	0.076(0.019,0.132)**	0.032(−0.023,0.086)
	*Model*2	0.199(0.134,0.264)***	0.108(0.047,0.168)***
	*Model*3	0.203(0.137,0.269)***	0.114(0.052,0.175)***
≥2 h	*Model*1	0.216(0.145,0.288)***	0.121(0.052,0.191)***
	*Model*2	0.277(0.197,0.357)***	0.126(0.051,0.202)**
	*Model*3	0.284(0.203,0.364)***	0.135(0.059,0.211)***

Model 1: conventional linear regression analyses, controlling for the confounders including time-invariant covariates and time-varying covariates at wave 1, and the other two sleep variables. Model 2: used stabilized inverse probability attrition weighted (IPTW). Model 3: Model 2 further added the other two sleep variables as the time-varying covariates to calculate adjusted stabilized IPTW. **p < 0.01. ***p < 0.001.

## Discussion

Using IPTWs in marginal structural model analyses, we found that accumulated waves of sleep disturbance and social jetlag in adolescence were significantly associated with an increase of cigarette use in young adulthood. Accumulated social jetlag—but not sleep disturbance—was also associated with an increase of alcohol use in adulthood. Accumulated waves of short sleep were not associated with a later increase of alcohol use unless it was less than 6 h of sleep per night; however, in our sample, short sleep was associated with less likelihood of cigarette smoking at age 21. With careful consideration of time-varying confounders and the temporal ordering of relationships, our study showed that not all unhealthy sleep practices were associated with later substance use in Taiwanese adolescents.

We found consistent evidence that cumulative social jetlag was associated with increased cigarette and alcohol use, which is consistent with the literature (O’Brien and Mindell, 2005; Pasch et al., 2010). One of the reasons may be peer influence. Social network analysis has demonstrated that both poor sleep behavior and substance use behavior can be spread in adolescents’ networks and that the two behaviors are correlated (Mednick et al., 2010). There may be social activities in peer groups that affect both adolescents’ sleep schedules on weekends and their smoking and drinking behaviors such as hanging out in entertainment venues past average bedtime and substance use with peers. However, more studies were needed to verify whether sleep behavior and substance use behavior can be spread in adolescents in different samples. It may also be due to the biological mechanism: the disruption of adolescent’s circadian rhythm may predispose them to more alcohol and cigarette use by altering the reward function in the brain development (Hasler et al., 2015). Social jetlag may negatively affect an individual’s endocrine and behavioral risk profile such as being more physically inactive or having an increased resting heart rate (Rutters et al., 2014), which may later be associated with increased depression and substance use. Intervening for social jetlag in adolescent populations is likely to reduce the likelihood of substance use in young adulthood.

The significant impact of short sleep on the decrease of cigarette use is a major difference in our sample that contrasts with the literature. Previous studies have mostly demonstrated an association opposite of our findings (McKnight-Eily et al., 2011). This may be explained by the influence of sleep insufficiency on cognition, resulting in the increase of vulnerability to peer pressure (O’Brien and Mindell, 2005), and the correlation between poor sleep practices and substance use by adolescents’ social networks and shared lifestyles (Mednick et al., 2010). However, the cause of short sleep may be different in an Asian context compared to Western culture and may be a result of a culture with a high priority for academic achievements rooted in Confucianism (Huang and Gove, 2015). Cultural differences in bedtime were noted in a review on worldwide sleep practices (Gradisar et al., 2011). Asian adolescents have significantly later bedtimes than European or North American adolescents (Gradisar et al., 2011). Reasons for restricted sleep time in school days in the Taiwan context could be for academic purposes. The emphasis on education in the society may influence the loading of homework and the attitude of parents to encourage spending more time on school work than in sleep. Hence, the mechanisms linking short sleep and smoking may be different from the Western context. It was hard to differentiate the academic-related factor when thinking about reasons why adolescents had short sleep in our dataset, such as spending lots of time studying instead of going to bed early. Future studies that include detailed reasons for short sleep in adolescents can further test the mediating effect of these reasons.

Our results showed different findings between conventional regression analysis and analysis using marginal structural models, such as in sleep disturbance; the significant effect in conventional regression analysis using the marginal structural model no longer exists. We used marginal structural models because conventional methods may give biased effect estimates when exposure affects a confounder or when exposure both affects and is affected by the study outcome, and also because marginal structural models have the ability to handle time-varying confounding and censoring (Robins et al., 2000). We interpreted the results of marginal structural models, instead of conventional models to avoid the above bias. One systematic review examined the publications in which marginal structural models and conventional analyses were used and found that few of the analyses (11%) showed opposite results between marginal structural models and conventional models. Among the rest of the studies that did not show opposite results, 40% showed that the marginal structural estimate differed by at least 20% from the conventional estimate on the usual scale (Suarez et al., 2011). Our paper is in the latter category. Our study showed that the standard errors of the marginal structural models associations are greater than the conventional standard errors, consistent with the findings of the review (Suarez et al., 2011). This increase in standard error has been described as a trade-off between bias and precision (Cole and Hernán, 2008).

Our findings should be interpreted considering the following limitations. First, other unmeasured confounders may exist. We focused a lot on individual level factors. Although we included peer drinking/smoking status as a time-varying confounder, there may be more relational or socio-environmental factors to be considered, such as a peer network that is associated with both sleep behavior and substance use because of shared lifestyles, induction, or their social behaviors. Other psychiatric conditions also likely impact both sleep and substance use, such as anxiety and attention deficit hyperactivity disorder. Second, sleep practice measurements were self-reported in our study and adolescents were only asked to report a time in general, which might have resulted in recall bias and social desirability bias. Objective measurement should be used in future studies, such as actigraphy or other validated wearable devices to better capture timing for going to bed and waking up. Similarly, since alcohol drinking and cigarette smoking behaviors were also self-reported, it is possible that these behaviors were underreported.

This study adds to the understanding of the longitudinal impact of three different dimensions of poor sleep practice in adolescence on substance use using a large sample size with a long follow-up that spanned 9 years in the adolescents’ life course. We highlighted that the effect of cumulative insufficient sleep on smoking and drinking may operate by different biological and social mechanisms, and that the cultural context should be taken into consideration. The use of marginal structural models provided an opportunity to evaluate the impact of insufficient sleep as time-dependent variables in the presence of time-dependent confounders, such as depression, which are affected by prior insufficient sleep. Mindfulness-based interventions and cognitive-behavioral therapy were effective to improve problematic sleep practices among adolescents (Blake et al., 2019). Interventions that aim to reduce the likelihood of substance use in young adulthood may consider incorporating such techniques to improve sleep practices in adolescents, particularly the discrepancy between bedtimes on school days and weekends and better quality of sleep.

## Data Availability Statement

The datasets for this study can be found: https://srda.sinica.edu.tw/index_en.php.

## Ethics Statement

The studies involving human participants were reviewed and approved by Institutional Review Board at the National Cheng Kung University in Taiwan (B-ER-107-028). Written informed consent to participate in this study was provided by the participants’ legal guardian/next of kin.

## Author Contributions

CS and C-YH conceptualized the structure of the study. C-YH conducted the formal analysis. C-YH and CS wrote the manuscript. S-HL monitored and validated the process of data analysis. M-CT and TY reviewed and edited the manuscript. CS, S-HL, M-CT, and TY acquired research funding.

## Conflict of Interest

The authors declare that the research was conducted in the absence of any commercial or financial relationships that could be construed as a potential conflict of interest.
